# Is the Additional Effort for an Intraoperative CT Scan Justified for Distal Radius Fracture Fixations? A Comparative Clinical Feasibility Study

**DOI:** 10.3390/jcm9072254

**Published:** 2020-07-16

**Authors:** Sascha Halvachizadeh, Till Berk, Alexander Pieringer, Emanuael Ried, Florian Hess, Roman Pfeifer, Hans-Christoph Pape, Florin Allemann

**Affiliations:** 1 Department of Trauma, University Hospital Zurich, Raemistrasse 100, 8091 Zurich, Switzerland; Till.Berk@usz.ch (T.B.); Alexander.Pieringer@usz.ch (A.P.); Emanuael.Ried@balgrist.ch (E.R.); Roman.Pfeifer@usz.ch (R.P.); Hans-Christoph.Pape@usz.ch (H.-C.P.); Florin.Allemann@usz.ch (F.A.); 2 Harald-Tscherne Research Laboratory, University Hospital Zurich, Sternwartstrasse 14, 8091 Zurich, Switzerland; 3 Department of Orthopaedic Surgery and Traumatology, Cantonal Hospital Frauenfeld, Pfaffenholzstrasse 4, 8501 Frauenfeld, Switzerland; florian.hess@stgag.ch

**Keywords:** intraoperative CT scan, distal radius fracture, O-arm radius fracture, intraoperative CT scan in trauma

## Abstract

Introduction: It is currently unclear whether the additional effort to perform an intraoperative computed tomography (CT) scan is justified for articular distal radius fractures (DRFs). The purpose of this study was to assess radiological, functional, and clinical outcomes after surgical treatment of distal radius fractures when using conventional fluoroscopy vs. intraoperative CT scans. Methods: Inclusion criteria: Surgical treatment of DRF between 1 January 2011 and 31 December 2011, age 18 and above. Group distribution: intraoperative conventional fluoroscopy (Group Conv) or intraoperative CT scans (Group CT). Exclusion criteria: Use of different image intensifier devices or incomplete data. DRF classification according to the Arbeitsgemeinschaft für Osteosynthesefragen (AO) classification. Outcome variables included requirement of revision surgeries, duration of surgery, absorbed radiation dose, and requirement of additional CT scans during hospitalization. Results: A total of 187 patients were included (Group Conv *n* = 96 (51.3%), Group CT *n* = 91 (48.7%)). AO Classification: Type A fractures *n* = 40 (50%) in Group Conv vs. *n* = 16 (17.6%) in Group CT, *p* < 0.001; Type B: 10 (10.4%) vs. 11 (12.1%), not significant (n.s.); Type C: 38 (39.6%) vs. 64 (70.3%), *p* < 0.001. In Group Conv, four (4.2%) patients required revision surgeries within 6 months, but in Group CT no revision surgery was required. The CT scan led to an intraoperative screw exchange/reposition in 23 (25.3%) cases. The duration of the initial surgery (81.7 ± 46.4 min vs. 90.1 ± 43.6 min, n.s.) was comparable. The radiation dose was significantly higher in Group CT (6.9 ± 1.3 vs. 2.8 ± 7.8 mGy, *p* < 0.001). In Group Conv, 11 (11.5%) patients required additional CT scans during hospitalization. Conclusion: The usage of intraoperative CT was associated with improved reduction and more adequate positioning of screws postoperatively with comparable durations of surgery. Despite increased efforts by utilizing the intraoperative CT scan, the decrease in reoperations may justify its use.

## 1. Introduction

Distal radius fractures (DRFs) represent the most common fractures of the upper extremity in adults (17.5%) [1]. Exact restoration of the anatomy and correct screw placement in the relevant fragment can be challenging, especially if the posterior articular surface is involved, or in cases of severe impaction [2]. These cases require revision surgery and the outcome is unfavorable (decreased range of motion, later wrist fusion) [3]. With conventional intraoperative imaging, incomplete reduction and inadequate screw placements have been reported [4]. The use of conventional fluoroscopy during surgery provides limited information, especially in complex DRFs [5]. Imaging of DRFs may include computed tomography (CT), especially in complex intra-articular fractures. The CT scan is a well-established imaging method that has proven diagnostic value [6]. To date, the majority of CT studies, however, are obtained for diagnostic reasons and for classification of injuries [7,8]. Advancements in intraoperative imaging, including mobile CT scans, have improved intraoperative image quality and allowed for immediate control of the osteosynthesis [9]. CT-based navigations have been used to increase the accuracy of screw and plate positioning during open reduction internal fixation (ORIF) of DRFs [10].

There continues to be a lack of studies that investigate the effects of intraoperative CT scans on the treatment of DRFs. The objective of this study was to compare surgical, radiological, and functional outcomes after operative treatment of DRFs with and without the use of intraoperative CT scans. We therefore aimed to answer the following questions:

- Is the use of intraoperative CT scans associated with increased surgical time and radiation dosage?
- Does an intraoperative CT scan lead to intraoperative revisions that may prevent reoperations?
- Does the intraoperative CT scan reduce the requirement of secondary CT scans during hospitalization?

## 2. Methods

This study was conducted with the approval of the cantonal ethical committee (Kantonale Ethikkommission, KEK, Zürich) and the institutional review board (IRB) under the license numbers 2019-01957 and 2018-00146.

### 2.1. Study Design and Study Population

This retrospective comparative study was performed among patients treated between 1 January 2011 and 31 December 2018, and was conducted in compliance with the Strengthening the Reporting of Observational studies in Epidemiology (STROBE) guidelines [11].

The inclusion criteria were as follows: patients aged 18 years and over who required surgical treatment of a distal radius fracture at one academic Level 1 trauma center.

Exclusion criteria: patients with incomplete data sets, missing radiation values, usage of other imaging techniques/devices, reoperations, open fractures, or more than one surgical intervention during the same session.

All patients were treated by a variable angle-locked compression plate (VA-LCP) and a two-column volar distal radius plate 2.4 (VA-LCP 2.4 mm, DePuy Synthes, 4436 Oberdorf, Switzerland).

The follow-up period was six months after surgery. Our standardized follow-up foresees 2 weeks, 6 weeks, 12 weeks, and 6 months of post-operative clinical and radiological examination.

### 2.2. Group Distribution and Intraoperative Radiologic Protocol

Intraoperative imaging defined the stratification of patients: conventional fluoroscopy (Group Conv) or additional intraoperative CT scans using the O-arm (O-arm; Medtronic, Minneapolis, MN) with standard configurations for extremity scans (Group CT).

### 2.3. Protocol for Intraoperative CT Use

According to our in-house protocol, an intraoperative CT scan has been used in all Type C fractures since 2015. After initial reduction, a fluoroscopic assessment of the plate and screw position is performed. The final assessment is then performed by a CT scan. If correction is required, a second CT scan is added after revision.

### 2.4. Definitions and Outcome Parameters

All DRFs were classified according to the Arbeitsgemeinschaft für Osteosynthesefragen (AO) classification [12]. Surgical factors: fracture classification with associated intraoperative imaging technique, duration of surgery, revision rate intraoperatively, and revision surgeries within six months after initial treatment. These data were retrieved from the radiological statement prior to surgery and the surgical report.

The radiological outcome included intraoperative radiation doses and the requirement of secondary CT scans after surgery during hospitalization. These data are automatically calculated by the imaging intensifier device (conventional fluoroscopy or intraoperative CT scan) and electronically sent to the patient’s chart.

During re-examination, the senior author (F.A.) measured the range of motion (ROM) in person to minimize inter-observer variability. The ROM was measured in six degrees of freedom: dorsal extension, palmar flexion, radial abduction, ulnar abduction, pronation, and supination.

A potential confounder was the fracture classification according to AO [13]. Therefore, the analysis included additional stratification according to the AO classification. The study size is based on the maximal available data set, according the inclusion and exclusion criteria.

### 2.5. Statistical Analysis

Continuous variables are summarized as mean and standard deviation, and categorical variables as number and percentage. Group comparison of two groups was performed using Student’s *t*-test for continuous variables, and the Pearson chi-squared test for discrete variables. ANOVA was performed to compare groups of more than two partners. Patients were stratified according to the imaging technique during surgery, and according to the fracture classification to control for confounding. Statistical significance was assumed at an alpha of 5% (*p* < 0.05). All analyses were performed using R (R Core Team (2019). R: a language and environment for statistical computing. R Foundation for Statistical Computing, Vienna, Austria. URL https://www.R-project.org/).

## 3. Results

We included 187 patients (mean age 55.1 years (±19.4 y)), among which 131 (70.1%) were females. In all patients, an acute fracture was the reason for fixation. The distribution according to the AO classification was as follows: Type A fracture (*n* = 64, 34.2%), Type B fracture (*n* = 21, 11.2%), Type C fracture (*n* = 102, 54.5%).

### 3.1. Patient Demographics

Group Conv (*n* = 96, 51.3%) and Group CT (*n* = 91, 48.7%) were comparable in age, gender distribution, body mass index (BMI), length of stay, and duration until returning to work. The incidence of Type A fractures in the Conv Group was 50% vs. 17.6% in the CT Group (*p* < 0.001). In Group CT, the leading fracture was Type C fractures, with 70.3% vs. 39.6% in the Conv Group (*p* < 0.001) (Table 1).

### 3.2. Intraoperative Revisions and Revision Surgeries

In Group Conv, 11 patients (11.5%) required a secondary CT scan after surgery. Out of these, four patients (36.4%) required revision surgeries: one case required a revision surgery due to intra-articular screw positioning, two cases required revision due to a malreduced fracture, and one case required a secondary stabilization of the distal radio-ulnar joint. None of the patients in Group CT required revision surgeries within six months. However, in 23 cases (25.3%) the CT scan revealed inadequate reduction, or inadequate implant position, that led to intraoperative revision.

### 3.3. Duration of Surgery and Radiation Dosage

Group Conv and Group CT were comparable in duration of surgery (95%CI 21.4 to 4.6, *p* = 0.21). The intraoperative radiation dose was significantly lower in Group Conv compared with Group CT (2.8 ± 7.8 mGy vs. 6.9 ± 1.3, *p* < 0.001). Group Conv required secondary CT scans after surgery during hospitalization in 11.5% of cases (Table 2). The radiation dosage of a secondary CT scan was significantly higher in Group Conv compared with the total intraoperative radiation dosage of Group CT (8.3 ± 2.5 mGy vs. 6.9 ± 1.3 mGy, 95%CI 0.4 to 3.2, *p* = 0.02).

### 3.4. Range of Motion

**Figure 1:**
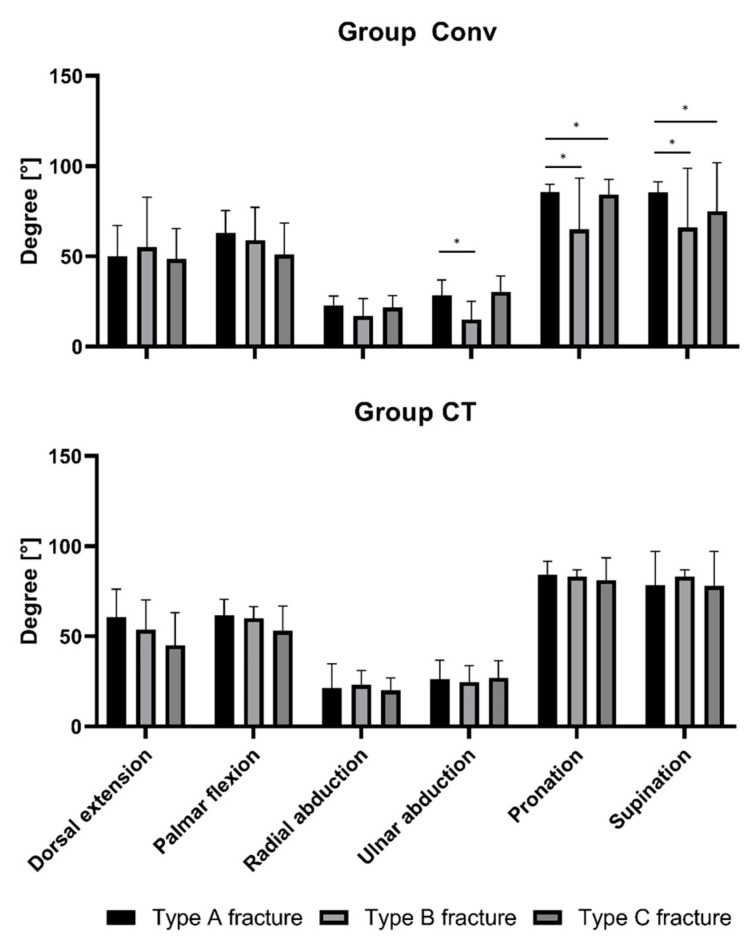
Comparison of Range of Motion (ROM) at six months after initial surgery of Group CT and Group Conv. While there is a significant difference amongst the subgroups in Group Conv, the ROM appears to be independent of the severity of fracture in Group CT. Types of fracture are classified according to the AO classification; * = significant difference (*p* < 0.05).

In Group Conv: Ulnar abduction Type A 30.4 ± 8.9° vs. Type B 51.0 ± 10.0°, *p* = 0.0026; Pronation Type A 85.7 ± 4.3° vs. Type B 65.0 ± 28.3°, *p* < 0.001 and vs. Type C 82.1 ± 8.2, *p* = 0.03; and Supination Type A 85.6 ± 5.7° vs. Type B 66.0 ± 32.9° *p* = 0.001 and vs. Type C 75.1 ± 26.8, *p* = 0.004. Type A, Type B, and Type C fractures had comparable ROMs in Group CT (Figure 1).

## 4. Discussion

Intraoperative anatomic reduction has been known to be a cofactor for good results in intra-articular fractures and those adjacent to joints [12]. Recent advancements in radiological imaging and improvement of CT technologies have led to an increased use of CT scans intraoperatively to guide and facilitate surgical interventions [14,15,16]. Currently, spine surgeons routinely use intraoperative CT scans [17,18]. Reports about the use for intra-articular fractures of the extremity are sparse [5]. In distal radius fractures, the variable anatomy and limited ability to visualize the dorsal aspect of the fracture’s articular surface represents a special challenge during surgical fixation of these fractures [19].

Our study provides the following main results:

1. The use of an intraoperative CT scan did not increase duration of surgery.
2. Intraoperative correction of screw positioning was performed in 25.3% of cases in Group CT. No further revision surgery was required.
3. Secondary CT scans were required in 11.5% of cases where no intraoperative CT was performed, resulting in an increased cumulative radiation dosage and revision surgeries in 36.4% of cases.

According to our data, the use of an intraoperative CT scan did not affect the overall duration of surgery. The time required for an intraoperative CT scan was shown to be 3 to 5 minutes during surgical correction of scoliosis without significant prolongation of the operation time [20]. During surgery of a DRF, the use of an intraoperative CT scan might take 6.7 ± 1.8 (range 4.3–13.3) minutes [5]. Previous authors have suggested that the duration of surgery may be less dependent on the imaging technique (fluoroscopy, CT scan) and more dependent on the experience of the surgeon, or the indication for surgery [21].

In a laboratory study with cadavers, no significant difference in breach rate between CT scans and fluoroscopy was observed during pedicle screw insertion [22]. Another study concluded that the increased radiation dose after the use of an intraoperative CT scan with an image-guided system was acceptable, given the benefits of CT imaging [23].

The present study indicates that the intraoperative use of CT is associated with higher levels of intraoperative radiation. During the postoperative course, some patients of Group Conv also required CT, which leads to a lesser sustained total radiation exposure. Another study indicates that during surgical treatment of DRFs, radiation exposure from fluoroscopy was 14.1 ± 15.4 cGycm^2^ compared with 19.3 ± 16.9 cGycm^2^ in cases where an additional CT scan was used, representing an increase of 36.9% [5]. This increase in radiation after additional usage of intraoperative CT scans is comparable with reported data that might range from 4.8 to 195% [5].

The use of CT scans has been suggested to improve visibility and assessment of intra-articular fractures [24]. Further, the use of postoperative CT scans after ORIF of DRFs provides more information regarding potential intra-articular placements of screws that otherwise would have been missed [25]. Our study revealed that in 11.5% of cases, a secondary CT scan was required that led to revision surgeries in 36.4% of cases. These secondary interventions could be prevented in cases that had an intraoperative CT scan. The quality of conventional fluoroscopy depends on projection and might increase false negative rates of intra-articular screw placement or poorly reduced fractures [26]. The use of intraoperative CT scans has been suggested to improve the assessment of screw placement and the quality of articular reduction independent of the user and projection, potentially decreasing the revision rate.

We are aware of several potential drawbacks. The sample size might introduce a type 2 error. However, based on completeness of data, especially radiologic measures, we believe that this sample size is sufficient. Further, the measurement of ROM might introduce some degree of variability. However, all ROMs were measured by one author (F.A.) to minimize variability. One might argue that the homogeneity of the study population might suffer based on a 7-year inclusion period. Yet, we believe that the inclusion of patients prior to the availability of intraoperative CT scans (2015) reduces confounding and selection bias, and that might increase confidence in our conclusion. The requirements for a secondary CT scan include insufficient information provided by fluoroscopy, pain, and discomfort. Since the intraoperative CT resulted in 25.3% of cases needing revision and the secondary CT scan in 36.4% of cases, we believe that the additional information given by the CT might improve the outcome of the patient. In addition, this is not a prospective study categorized according to severity of fractures. However, we feel that the automatized documentation of radiation dosages and reporting of reoperation rates and intraoperative revisions allows us to draw the following conclusions.

## 5. Conclusions

In our series, an intraoperative CT scan was not associated with an increased duration of surgery. We observed a decrease in the revision rate and improved screw positioning after the use of an intraoperative CT scan. We feel that these findings support the limited use of intraoperative CT scans in patients with complex fractures of the radius.

## Figures and Tables

**Table 1:** Patient demographics.

	Group Conv	Group CT	*p*-Value
*n*	96	91	
Age (years), mean (SD)	54.8 (19.7)	55.4 (19.1)	n.s.
Female gender, *n* (%)	67 (69.8)	64 (70.3)	n.s.
BMI (kg/m^2^), mean (SD)	24.6 (4.4)	26.5 (17.3)	n.s.
AO classification, *n* (%)			
A	48 (50.0)	16 (17.6)	<0.001
B	10 (10.4)	11 (12.1)	n.s.
C	38 (39.6)	64 (70.3)	<0.001
Length of stay (days), mean (SD)	7.0 (8.8)	7.9 (6.6)	n.s.
Weeks until return to work, mean (SD)	5.9 (4.4)	6.2 (4.5)	n.s.

*n* = Number of patients; SD = Standard Deviation; n.s. = Not Significant (*p* > 0.05); BMI = Body Mass Index; AO = Arbeitsgemeinschaft für Osteosynthesefragen.

**Table 2:** Radiographic and intraoperative data.

	Group Conv	Group CT	*p*-Value
*n*	96	91	
Revision Surgeries within 6 months, *n* (%)	4 (4.2)	0 (0.0)	n.s.
Intraoperative revision based on CT, *n* (%)	Not documented	23 (25.3%)	NA
Duration of surgery (minutes), mean (SD)	81.7 (46.4)	90.1 (43.6)	n.s.
Intraoperative Radiation Dose (mGy), mean (SD)	2.8 (7.8)	6.9 (1.3)	<0.001
Requirement of secondary CT scan, *n* (%)	11 (11.5%)	0 (0%)	<0.001
Radiation Dose of secondary CT scan (mGy), mean (SD)	8.3 (2.5)	NA	

AO = Arbeitsgemeinschaft für Osteosynthesefragen; *n* = Number of patients; SD = Standard Deviation; mGy = milligray; n.s. = Not Significant (*p* > 0.05).
